# Direct conversion of underutilized tropical fruit wastes to 5-hydroxymethylfurfural using a strongly acidic deep eutectic solvent: mechanistic study, renewable extraction, and life cycle assessment

**DOI:** 10.1039/d6ra01120a

**Published:** 2026-03-24

**Authors:** Quang Tam Huynh, Udomsap Jaitham, Hai Nguyen Tran

**Affiliations:** a Institute of Fundamental and Applied Sciences, Duy Tan University Ho Chi Minh City 70000 Vietnam huynhquangtam@dtu.edu.vn; b School of Health Sciences Research, Research Institute for Health Sciences, Chiang Mai University Chiang Mai 50200 Thailand; c Faculty of Environment and Chemical Engineering, Duy Tan University Da Nang 50000 Vietnam

## Abstract

A highly efficient and sustainable route for the direct production of 5-hydroxymethylfurfural (5-HMF) from untreated lignocellulosic biomass is reported using a novel Brønsted–Lewis deep eutectic solvent (DES) based on betaine hydrochloride and AlCl_3_ (HBetCl:AlCl_3_), which functions simultaneously as a solvent and catalyst. This research demonstrates the inaugural utilization of a betaine-derived chloroaluminate deep eutectic solvent (DES) for the synthesis of 5-HMF, offering remarkably robust and adjustable dual acidity without necessitating biomass pretreatment, supplementary mineral acids, or other organic solvents. Under mild conditions of 135 °C, 150 min, 10 vol% water, HBetCl : AlCl_3_ = 1 : 0.5, jackfruit rind produced an excellent 5-HMF yield of 66.42 ± 0.15 mol%. Density functional theory (DFT) simulations show a strong synergistic effect between Brønsted and Lewis acidic sites. Chloroaluminate species lower the glucose-to-fructose isomerization barrier from more than 110 kJ mol^−1^ to 46 kJ mol^−1^, which is also supported by kinetic modeling. The process also showed that bio-based 2-methyltetrahydrofuran (2-MeTHF) could be used to recycle DES effectively for 5-HMF extraction. The DES kept its high catalytic performance over several cycles, with little AlCl_3_ leaching and good phase separation. It retained 65.23% yield after the first recycle and stayed active for five runs in a row. Furthermore, a cautious cradle-to-gate life cycle evaluation also shows that 5-HMF has a global warming potential of 15.6 kg CO_2_ eq. per kg, which is a good starting point for sustainable biomass valorization.

## Abstract 2

A sustainable route for producing 5-hydroxymethylfurfural from untreated lignocellulosic biomass is developed using a Brønsted–Lewis superacidic deep eutectic solvent (HBetCl:AlCl_3_), acting as both solvent and catalyst.

## Graphical abstract

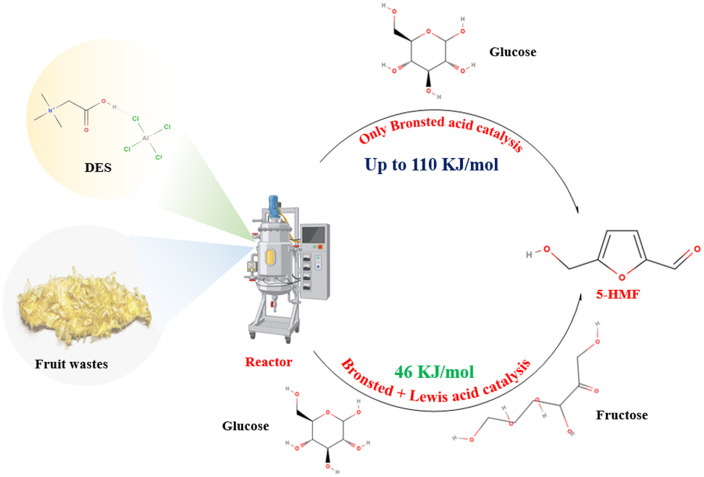

## 1. Introduction

Tropical fruits are recognized as nutrient-dense sources of essential vitamins and minerals, serving as vital dietary supplements for both human populations and livestock.^1^ Economically important tropical tree species are mostly native to South and Southeast Asia, particularly cultivated in regions such as India, Thailand, Vietnam, and Indonesia.^2,3^ In recent years, their agricultural presence has expanded into other parts of the world, such as South America and northern Australia.^4^ Although the flesh of them is the main edible portion, approximately 30% to 60% of the fruit's total weight consists of inedible components, such as the rag, seeds, rind or peel, bulb remnants, and inner core, which are typically discarded as biological waste, contributing to overall agro-industrial residue.^1,5^ Among the various by-products, the rind or peel is one of the promising lignocellulosic biomasses.^6^ Its structural composition, comprising high cellulose, moderate hemicellulose, and low lignin,^7,8^ provides a carbohydrate-rich matrix amenable to a promising renewable substrate for the sustainable production of high-value biochemicals and biofuels within emerging biorefinery frameworks. This sustainable valorization strategy aligns closely with circular economy principles by transforming abundant, discarded carbon-rich tropical fruit wastes into high-value platform chemicals, while contributing to the United Nations Sustainable Development Goals, particularly Goal 9 (Industry, Innovation and Infrastructure) and Goal 12 (Responsible Consumption and Production).

5-Hydroxymethylfurfural (5-HMF), a pivotal intermediate derived from biomass, has gained remarkable attention as a foundation for developing renewable chemicals and materials. Its unique molecular framework, featuring an aldehyde and two hydroxyl groups, enables a wide range of chemical transformations, making it one of the most adaptable compounds in green chemistry.^9^ Notably, the hydrogenation of 5-HMF produces 2,5-dimethylfuran (DMF), a promising next-generation biofuel with superior energy density and hydrophobicity compared to ethanol.^10^ Through oxidation, 5-HMF can be converted into 2,5-furandicarboxylic acid (FDCA),^11^ a renewable analog of terephthalic acid that serves as a monomer for polyethylene furanoate (PEF), a biobased polyester exhibiting enhanced barrier and mechanical properties relative to polyethylene terephthalate (PET).^12^ Furthermore, 5-HMF can be chemically transformed into a range of other chemicals including levulinic acid, a platform chemical with uses in the production of polymers, pharmaceuticals, and agrochemicals.^13^ Given its versatility and sustainability, 5-HMF continues to attract significant interest from both academia and industry, making it a cornerstone of the emerging bio-economy.

C6 carbon sugars, including fructose, glucose, or mannose, have been favored substrates for 5-HMF production due to their ketose nature.^14^ However, their relatively high market cost challenges economic viability, especially on an industrial scale.^15^ Consequently, the most promising avenue for sustainable and cost-effective 5-HMF production appears to be using raw agricultural wastes, such as rice straw, sugarcane bagasse, and just wastes. This approach aligns seamlessly with green chemistry principles, allowing for converting this bountiful material into valuable chemicals like 5-HMF while minimizing environmental impact and advancing sustainability.^16^ Nonetheless, harnessing the potential of biomass presents technological challenges due to its inherent complexity, necessitating the development and fine-tuning of advanced conversion processes.

Even though almost every 5-HMF synthesis process from biomass-derived sugars requires suitable catalytic reagents to achieve high yield and selectivity, they have drawbacks that limit their efficiency and sustainability.^17,18^ For instance, homogeneous catalysts, such as mineral or organic,^11,19^ can cause the degradation of furfurals and the formation of humins and other byproducts, which complicate the separation and recycling processes. On the other hand, heterogeneous catalysts, such as metal–organic frameworks (MOFs)^20^ or metal phosphates (MPs),^21^ can offer a more practical solution as they are easier to separate and recycle. However, these catalysts are also prone to deactivation by the accumulation of byproducts and humins on their surface, which block their active sites, reducing their activity.^22^ Furthermore, different phase reactions result in less connection among reaction chemicals, leading to less selectivity.^11^ Therefore, there is a need to develop novel and improved methods for synthesizing 5-HMF from biomass-derived sugars to overcome these challenges.

Deep eutectic solvents (DESs) have recently been attracting increased interest. Characterized by their low melting temperatures compared to pure substances, DESs consist of a hydrogen bond acceptor (HBA) and one or several corresponding donors (HBD).^23^ Among these, Lewis–Brønsted acidic DESs have been widely explored for carbohydrate dehydration to 5-HMF, due to their dual functionality as both solvent and catalyst.^24^ For instance, choline chloride-based DESs paired with metal halides such as CrCl_3_ or FeCl_3_ have demonstrated moderate 5-HMF yields from glucose^25^ and fructose,^26^ yet suffer from poor selectivity due to concurrent rehydration of 5-HMF to levulinic acid and humin formation.^11^ Ionic liquids (ILs) such as [BMIM]Cl and [EMIM]Br have similarly been reported as effective media for cellulose dissolution and dehydration,^27^ but their high synthesis cost, and difficult recyclability limit their practical scalability. Recently, DES-based betaine is a relatively new and emerging topic of research with some advantages over conventional solvents, such as low lower cost, wider availability, high thermal stability, and less toxicity.^28^ There are several studies that have explored the properties and applications of DES based betaine hydrochloride, such as CO_2_ capture,^28^ metal production,^29^ and enzyme extraction.^30^ However, there are also some challenges and limitations that need to be addressed, such as the preparation, and recyclability of DES-based betaine. Therefore, DES-based betaine is promising and interesting research for biorefinery fields, but it still needs more development and innovation to become more popular and widely used.

Acid-based DESs, which exhibit dual functionality as solvents and catalysts, have a drawback: they tend to convert 5-HMF to levulinic acid and humic compounds, a less desirable product.^22^ In contrast, nonproton-polar organic solvents, such as dimethyl sulfoxide (DMSO), acetonitrile, tetrahydrofuran (THF), or dimethylformamide (DMF), can prevent this side reaction, but they may need additional catalysts or additives and make downstream processes more complex.^16,31–33^ Biphasic systems combining aqueous acid catalysts with organic extraction solvents have also been proposed to improve 5-HMF selectivity,^34^ but these require precise phase management and often rely on purified feedstocks such as fructose, glucose, or cellulose. Microwave-assisted systems have further been reported to improve reaction rates and selectivity,^35^ but their energy intensity and limited scalability constrain industrial applicability.

To overcome these limitations, a novel HBetCl:AlCl_3_-based DES system is proposed, which incorporates the merits of both Lewis and Brønsted acidity. The proposed HBetCl:AlCl_3_-based DES system offers three significant improvements: (i) strong synergistic Brønsted–Lewis acidity, which is achieved by the HBetCl:AlCl_3_ interaction, allowing for efficient catalyst performance without the need for an additional acid, (ii) direct conversion of untreated lignocellulosic waste, avoiding the need for feedstock fractionation and purification, as compared to conventional carbohydrate dehydration reactions, (iii) operation of the proposed process in a simple batch reactor under high pressure, as compared to literature systems, which often require microwave heating, biphasic systems, or other sophisticated configurations. This proposed method makes the conversion process easier by avoiding the use of any catalyst or additive. This novel approach not only overcomes the limitations of conventional DES/IL-based systems but also contributes to the concept of the circular economy by utilizing agricultural waste as a source of value-added chemicals.

## 2. Material and methodology

### 2.1. Reagents and biomass

Unless otherwise specified, all chemicals utilized in this study were commercially available and were not purified further. Betaine (C_5_H_12_C_l_NO_2_, 99%), and hydrochloric acid (HCl, 37%) were purchased from Thermo Scientific (Belgium, China). Anhydrous aluminum chloride (AlCl_3_, 99%), was obtained from Avantor, Inc. (Pennsylvania, US).

Various biomass, including jackfruit rind, banana peel, pineapple peel, and durian rind were collected in the southern region of Vietnam. About 500 grams of biomasses were cut into minute fragments and desiccated at 105 °C until a constant weight was achieved. The samples were then ground and sieved through a 100-mesh screen in preparation for subsequent component analyses.

### 2.2. Synthesis of deep eutectic solvents

As previously mentioned in the literature,^28^ the deep eutectic solvents (DESs) employed in the experiments of this work were created by physical mixing through two steps:

Step (1). Preparation of betainium chloride (HBetCl): betaine was placed in a dry, nitrogen-purged flask and cooled in an ice bath. Concentrated HCl (1.0 mol per mol betaine) was added dropwise under vigorous stirring, keeping the temperature below 50 °C. The resulting viscous melt was stirred for an additional 30–60 min and subsequently dried under vacuum at 40–50 °C overnight to remove residual water, yielding solid HBetCl.

Step (2). Formation of the DES: HBetCl and anhydrous AlCl_3_ were combined in a 1.0 : 0.30–0.50–0.70 molar ratio in a sealed, N_2_-blanketed flask. The mixture was gently heated to 60–70 °C and stirred until a clear, homogeneous liquid formed. No solvent or additional water was introduced at any stage. The final material was stored warm (40–50 °C) and transferred to a dry, airtight container under.

### 2.3. The conversion of jack fruit rind to 5-HMF

Fruit wastes (0.20 g) was added to anhydrous HBetCl : AlCl_3_ DES (5 mL; 1.0 : 0.35–0.70 mol mol^−1^) and 0.5 mL DI water in a sealed high-pressure glass reaction bottle, which was heated in an oil bath at 100–150 °C for 45–120 min. After reaction, the hot mixture was immediately filtered to remove solid residues. 2-Methyltetrahydrofuran (2-MeTHF, 10 mL) was added to the filtrate mixture containing HBetCl:AlCl_3_ DES, and the mixture was stirred at 80 °C for 20 min to extract 5-HMF. The system was cooled to room temperature, during which the DES phase containing the became highly viscous and partially solidified, allowing phase separation by decantation. The organic phase was collected and concentrated under reduced pressure at 40 °C (200–100 mbar) to a small residual volume, which was then subjected directly to recrystallization from cold 2-MeTHF (−25 °C) to obtain high pure 5-HMF.

All experiments were performed in triplicate and the reported values represent the average results with standard deviations.

### 2.4. Determination of the products

The obtained 5-HMF and furfural concentrations were determined by the high-performance liquid chromatography (HPLC, Hitachi CM5000, Japan) using the column Myghtysil RP-18 GP 5 µm 250 × 4.6 mm with the UV (wavelength 284 nm) detector and the column maintained at 40 °C, by pure water/acetonitrile (80 : 20) as a mobile phase flow rate 0.6 mL min^−1^.

The yield of 5-HMF was calculated on a molar basis as follows eqn (1):1
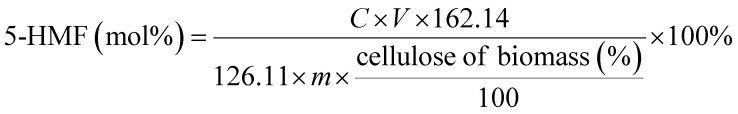
where, *C*: concentration of 5-HMF (g mL^−1^). *V*: volume of solvents after reaction (mL). 162.14 g mol^−1^ = molar mass of cellulose. 126.11 g mol^−1^ = molar mass of 5-HMF. *m*: weight of biomass loading.

### 2.5. Density functional theory (DFT)

Density functional theory (DFT) calculations were performed using the B3LYP functional,^36^ which incorporates three Becke exchange parameters. The 6-311++G(d,p) basis set was selected for its ability to reliably describe electronic and geometric properties. Frequency calculations were conducted to confirm the nature of each stationary point; that minima were verified by the absence of imaginary frequencies, while transition states were confirmed by a single imaginary frequency corresponding to the reaction coordinate. All reported energies are electronic energies at 298.15 K. Calculations were carried out using the Gaussian 16 software package.

### 2.6. Fourier transform infrared (FTIR) spectrophotometry

FTIR technique was used a Thermo Nicolet iS5 Fourier-transform infrared spectrometer from Thermo Fisher Scientific, which equipped with an ATR diamond crystal and operated with OMNIC software. To evaluate surface acidity, a small amount of pyridine vapor was introduced by placing a droplet of liquid pyridine in a sealed chamber together with the DES-coated ATR plate and allowing adsorption for 10 min. Excess pyridine was removed with a dry N_2_ flow before measurement. Spectra were recorded from 1800 to 1000 cm^−1^ with 4 cm^−1^ resolutions and 32 scans. The characteristic absorption bands at around 1540 cm^−1^ (Brønsted acid sites) and around 1450 cm^−1^ (Lewis acid sites) were used for acidity assignment.^37^

### 2.7. Nuclear magnetic resonance (NMR)

The ^27^Al NMR spectra were recorded on a Bruker Avance NMR Spectrometer operating at a resonance frequency of 160.15 MHz at 25 °C. Chemical shifts were referenced to an external standard consisting of 1.0 M Al(NO_3_)_3_ in D_2_O (0 ppm). The DES samples were analyzed directly without further modification.

### 2.8. Life cycle assessment

#### 2.8.1. Goal and scope

This cradle-to-gate attributional LCA evaluates the environmental performance of 5-HMF production from untreated jackfruit rind using the HBetCl:AlCl_3_ DES system. The goal is to quantify impacts, identify hotspots, and compare with literature benchmarks. The functional unit is 1 kg of 5-HMF. The system boundary includes biomass collection, DES synthesis, HMF synthesis, and extraction/separation by using bio-based 2-MeTHF.

#### 2.8.2. Life cycle inventory (LCI)

Primary data come from experimental results: feedstock composition, HBetCl : AlCl_3_ ratio (1 : 0.5), water (10 vol%), energy inputs, 66.42 mol% yield, and recyclability. Secondary data for upstream processes (betaine hydrochloride, AlCl_3_, electricity, diesel transport, 2-MeTHF) were sourced from Ecoinvent v3. Short-distance local transport (<100 km) was assumed for tropical waste collection. Inventory flows were scaled to the functional unit, with cut-off <1% of mass and energy.

#### 2.8.3. Life cycle impact assessment (LCIA)

The ReCiPe 2016 midpoint (hierarchist) method was used to characterize 18 impact categories, including global warming, ozone depletion, ecotoxicity, human toxicity, land use, resource scarcity, and water consumption. Characterization was performed using SimaPro LCA software with Ecoinvent-compatible databases. No endpoint assessment or weighting was applied.

#### 2.8.4. Interpretation

Contribution and hotspot analyses were conducted *via* percentage shares and absolute allocations. Uncertainties (*e.g.*, electricity mix, recycling efficiency, lab-to-scale extrapolation) were addressed qualitatively and through potential sensitivity scenarios.

This methodology follows ISO 14040/14044 for transparency and reproducibility in prospective biorefinery LCAs.^38^

## 3. Results and discussion

### 3.1. Biomass material composition analysis

The contents of lignin, cellulose, and hemicellulose from various biomass were obtained through the analysis, and the results of material components in this study are shown in Table 1.

**Table 1:** Biomass material compositions

Biomass	Cellulose (%)	Hemicellulose (%)	Lignin (%)	Extractives (%)
Jackfruit rind	28.56 ± 1.25	22.57 ± 0.23	2.46 ± 0.03	32.12 ± 0.03
Banana peel	22.12 ± 0.12	21.35 ± 0.26	14.23 ± 0.05	32.06 ± 0.12
Pineapple peel	26.18 ± 0.13	28.02 ± 0.03	6.42 ± 0.17	12.13 ± 0.08
Durian husk	52.10 ± 0.09	25.13 ± 0.25	13.23 ± 0.52	3.23 ± 0.23

The compositional profiles of the investigated fruit wastes highlight their strong potential as renewable feedstocks for biochemical synthesis. Jackfruit rind and pineapple peel exhibit balanced holocellulose contents of approximately 51% and 54%, respectively, combined with relatively low lignin levels, indicating high carbohydrate accessibility for the production of furan derivatives. Banana peel, despite its higher lignin content, contains substantial hemicellulose and extractives, suggesting suitability for the recovery of fermentable sugars, pectin-derived products, or nitrogen-containing biochemicals. Durian husk shows exceptionally high cellulose content at 52.10%, making it a promising precursor for glucose-derived chemicals, although its elevated lignin may require targeted processing. Overall, these results demonstrate that fruit wastes represent versatile and underutilized resources that can be selectively directed toward different biochemical pathways depending on their intrinsic composition.

### 3.2. Characteristics of DES

**Fig. 1:**
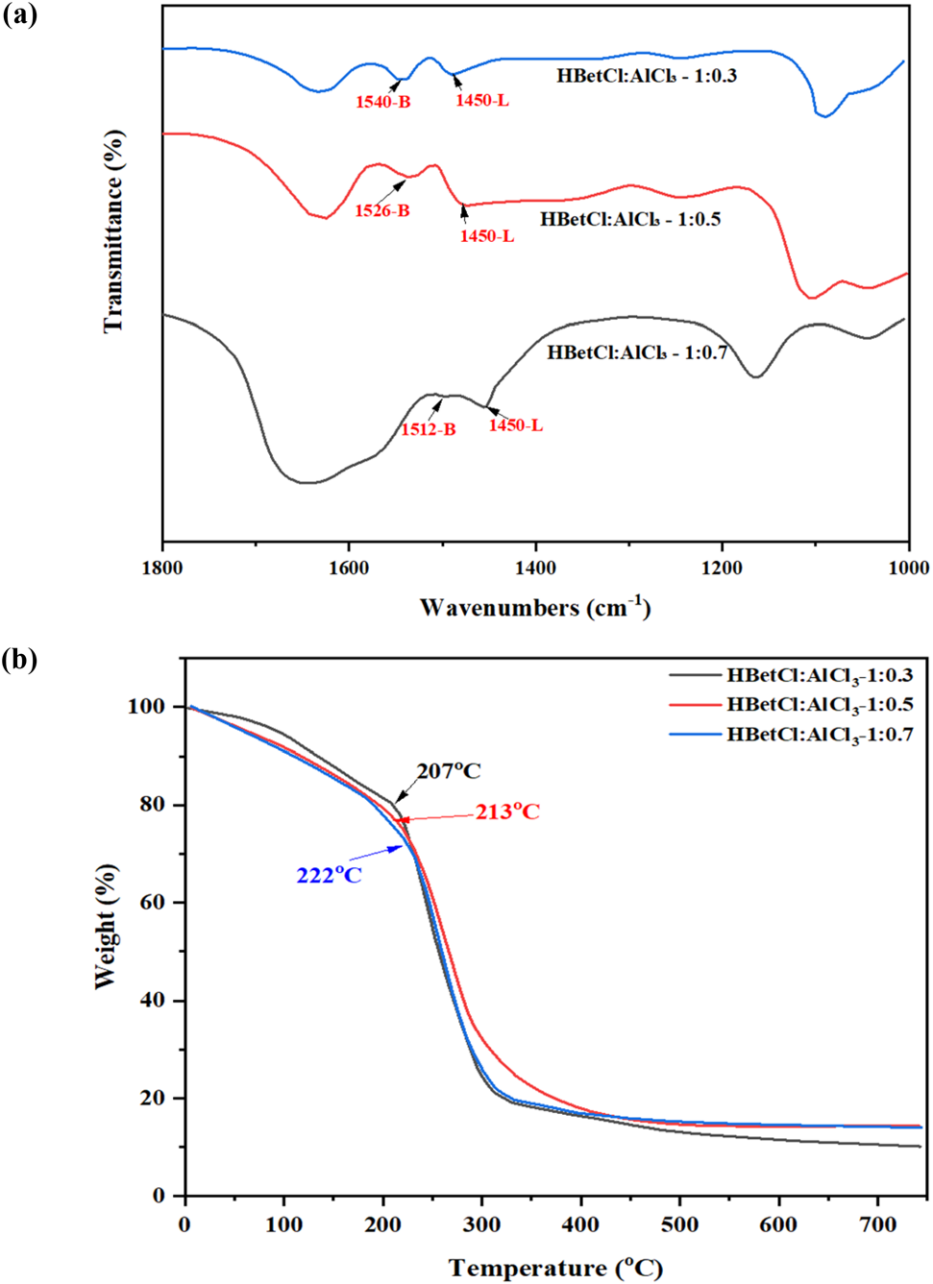
(a) FTIR spectra for Brønsted and Lewis acid analysis and (b) TG analysis.

The pyridine-adsorbed FTIR spectra of the HBetCl:AlCl_3_ DES as displayed in Fig. 1(a) clearly reveal the coexistence of both Brønsted and Lewis acid sites, and the relative strength of these sites varies systematically with the AlCl_3_ content.

Characteristic Brønsted acid bands, corresponding to protonated pyridine (PyH^+^), appear at 1540 cm^−1^ for DES 1 : 0.3, shift to 1526 cm^−1^ for DES 1 : 0.5, and decrease further to 1512 cm^−1^ in the AlCl_3_-rich 1 : 0.7 system.^39^ This gradual downshift indicates an increasingly stronger proton-donating ability as the AlCl_3_ ratio increases. This behavior is consistent with the formation of more acidic groups, including a partially protonated betainium moiety and a stronger hydrogen-bonding network, in the presence of higher equivalents of Lewis acid AlCl_3_. At the same time, Lewis acid sites, defined by the pyridine-Al coordination band at around 1450 cm^−1^,^37^ are observed across all DES components. The presence of this band confirms the presence of chloroaluminate anions, which act as Lewis acid sites capable of coordinating pyridine. Although the band positions remain similar in all samples, the relative intensity increases at DES 1 : 0.7, indicating a higher concentration of Lewis acid chloroaluminate species.

The thermal decomposition profiles of the HBetCl:AlCl_3_ as displayed in Fig. 1(b) exhibit a similar two-stage degradation pattern, with the major weight-loss event occurring between 180 and 222 °C. The onset of rapid mass loss shifts systematically with the AlCl_3_ content, revealing a clear relationship between composition and thermal stability. The 1 : 0.3 DES shows the earliest degradation onset at 207 °C, followed by the 1 : 0.5 DES at 213 °C, whereas the 1 : 0.7 DES displays the highest thermal stability with an onset near 222 °C. The improved stability at higher AlCl_3_ ratios can be attributed to the formation of more thermally robust chloroaluminate anions, especially AlCl_4_^−^ and Al_2_Cl_7_^−^, which reduce the volatility and suppress early decomposition of the protonated betainium species. Overall, the TGA results confirm that all three DES formulations are thermally stable well above 150 °C, validating their suitability for catalytic reactions such as biomass dehydration under typical operating temperatures.

### 3.3. Biomass conversion results in HBetCl:AlCl_3_ DESs

**Fig. 2:**
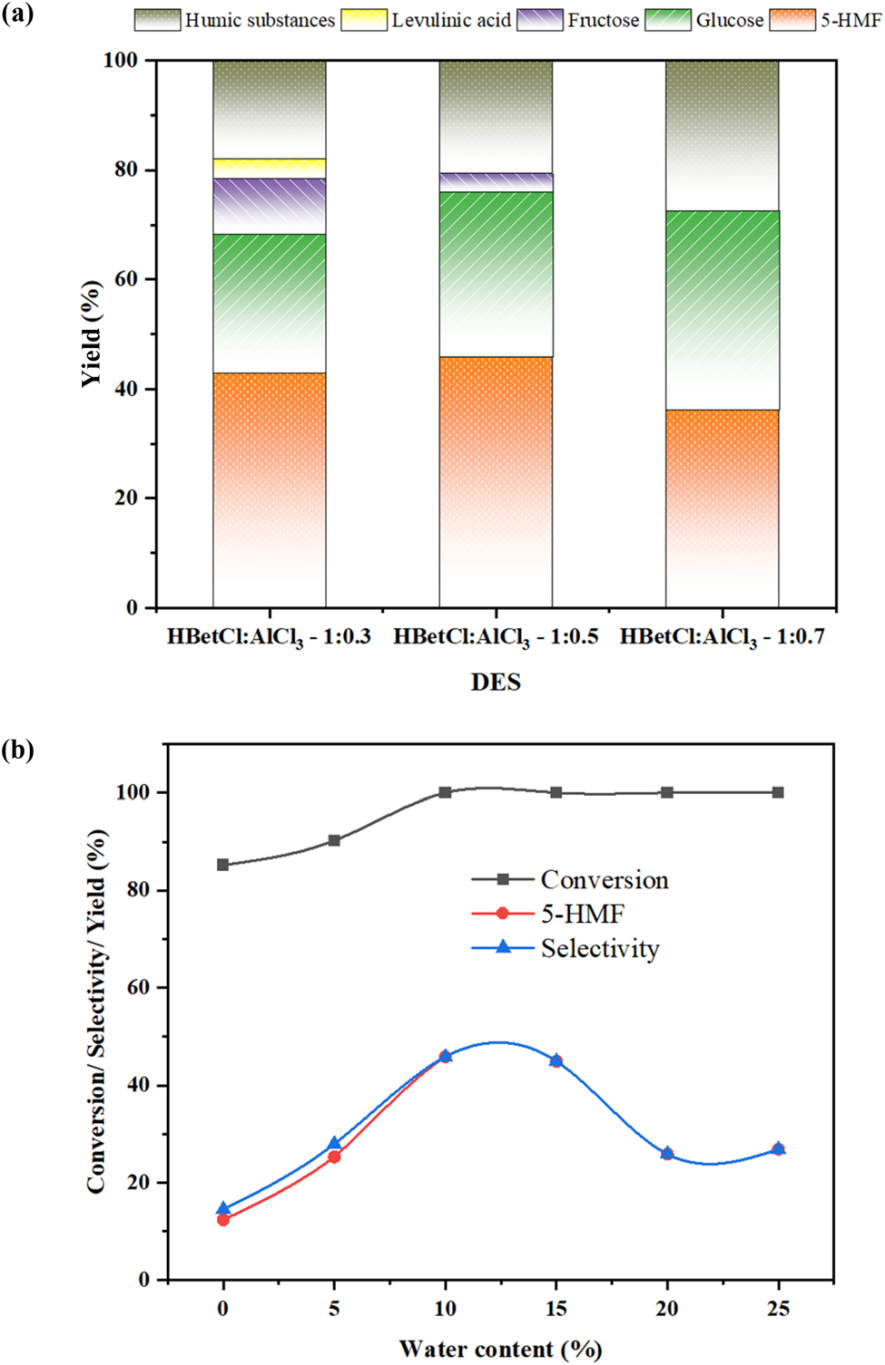
(a) The effect of different DESs in different solvents. Reaction condition: 200 mg of jackfruit rinds as the reaction substrate, 5 mL of DES, 0.5 mL DI water, and 120 °C heating in high pressure reactor in 1 hour. (b) The effect of different water content. Reaction condition: 200 mg of jackfruit rinds as the reaction substrate, 5 mL of DES, certain percentages of mL DI water, and 120 °C heating in high pressure reactor in 1 hour.

The catalytic performance of the HBetCl:AlCl_3_ DES shows a strong dependence on the AlCl_3_ content, reflecting the distinct balance between Brønsted and Lewis acidity within each formulation. Using 200 mg of jackfruit rind as the reaction substrate, 5 mL of DES, 0.5 mL DI water, and 120 °C heating in high pressure reactor in 1 hour. As seen in Fig. 2(a), the reaction between jackfruit rinds and different DESs gave different amounts of products depending on the ratios of HBetCl and AlCl_3_. The 1 : 0.3 and 1 : 0.5 DESs exhibit the highest 5-HMF yields, 43.01% and 45.91%, respectively, demonstrating that moderate acidity is most effective for promoting both glucose isomerization and subsequent fructose dehydration.

In contrast, the strongly Lewis-acidic 1 : 0.7 DES delivers a noticeably lower 5-HMF yield at 36.15% and retains a high proportion of unreacted glucose at 36.45%, with no detectable fructose. This aligns with the pyridine-FTIR results, which show intensified Lewis acidity at higher AlCl_3_ ratios. Excessive Lewis acidity likely leads to strong complexation between Al species and glucose, hindering glucose isomerization and slowing the overall conversion sequence. As a result, the reaction stalls at the early stages, reducing HMF formation.

The production of levulinic acid from a rehydration of 5-HMF occurs only in the 1 : 0.3 system around 3.5%, suggesting that weaker Lewis acidity allows some HMF to undergo secondary degradation. At higher AlCl_3_ levels (1 : 0.5 and 1 : 0.7), the DES binds water more strongly and stabilizes the catalytic environment, thereby suppressing 5-HMF rehydration completely. These results demonstrate that moderate acidity with 1 : 0.5 ratio achieves the optimal balance between sugar activation, and minimization of side-reactions.

The experimental data in Fig. 2(b) showing the effect of water content on cellulose conversion and 5-HMF yield is highly insightful for optimizing the reaction. Increasing the water content from 0% to 10% significantly improves cellulose conversion, reaching 100% at and above 10%. This initial increase confirms that water acts as a crucial reactant for the initial hydrolysis step, facilitating the breakdown of the polymer backbone. Concurrently, 5-HMF yield and selectivity rises sharply to its maximum of 45.89% at the same 10% water content. This indicates that a small, controlled amount of water is necessary not only for the initial depolymerization but also to effectively facilitate the subsequent dehydration steps leading from fructose to the desired 5-HMF product. Beyond the 10% threshold, the performance rapidly deteriorates, demonstrating a critical trade-off between hydrolysis and product degradation. While cellulose conversion remains at 100% the 5-HMF yield and selectivity drops significantly, falling to 25.86% at 20–25% of water content. This decline is directly explained by excessing water strongly favors the degradation of HMF into undesirable products, particularly levulinic acid and humic compounds. Overall, the reliable results establish that 10% of water content represents the optimal balance, maximizing cellulose breakdown while minimizing the solvent's ability to drive 5-HMF consumption through competing, water-sensitive side reactions.

### 3.4. Influence of process parameters

Response surface methodology (RSM) using a Box–Behnken design was employed to assess the effects of reaction time, reaction temperature, and DES dosage on 5-HMF production from jackfruit rind. The factor levels are listed in Table 2, and 17 randomized experimental runs were performed to avoid systematic bias. The full design matrix and responses are presented in Table 3. Eqn (2) was used to make predictions about the response. Multiple regression analysis was then applied to generate a second-order polynomial model describing the relationship between the coded variables and the 5-HMF yield.

2 Yield = 25.45 + 17.11 × *A* + 8.50 × *B* + 8.76 × *C* − 5.46 × *AB* + 2.01 × *AC* + 2.50 × *BC* + 12.44 × *A*^2^ + 0.11 × *B*^2^ − 4.72 × *C*^2^

Yield is the 5-HMF yield, and *A*, *B*, and *C* are the coded factors of the test variables time, temperature, and DES dosage, respectively.

**Table 2:** Experimental factors and levels used in Box–Behnken design

Independent variable units code	Units	Code	Level		
			−1	0	1
Reaction time	min	*A*	30	90	150
Reaction temperature	°C	*B*	100	125	150
DES dosages	mL	*C*	2	3.5	5

**Table 3:** The Box–Behnken design matrix for variables along with experimental and predicted values response

Run order	*A* (min)	*B* (°C)	*C* (mL)	5-HMF yield
				Experimental (%)
1	90 (0)	125 (0)	3.5 (0)	20.96
2	90 (0)	125 (0)	3.5 (0)	25.36
3	150 (+1)	125 (0)	5 (+1)	60.18
4	30 (−1)	125 (0)	2 (−1)	10.2
5	30 (−1)	125 (0)	5 (+1)	26.45
6	150 (+1)	125 (0)	2 (−1)	35.89
7	90 (0)	150 (+1)	2 (−1)	21.02
8	30 (−1)	150 (+1)	3.5 (0)	31.04
9	90 (0)	125 (0)	3.5 (0)	27.13
10	90 (0)	100 (−1)	5 (+1)	15.67
11	90 (0)	125 (0)	3.5 (0)	23.45
12	90 (0)	100 (−1)	2 (−1)	5.89
13	90 (0)	125 (0)	3.5 (0)	30.36
14	150 (+1)	100 (−1)	3.5 (0)	55.89
15	30 (−1)	100 (−1)	3.5 (0)	6.23
16	150 (+1)	150 (+1)	3.5 (0)	58.85
17	90 (0)	150 (+1)	5 (+1)	40.78

The ANOVA results in Table 4 show that the quadratic model is highly significant with a *p* value of 0.0001, indicating that the selected variables effectively describe the variation in 5-HMF yield. Reaction time exhibited the strongest linear effect with a *p* value lower than 0.0001, followed by reaction temperature with a *p* value of 0.0008 and DES dosage with a *p* value of 0.0006, confirming that all three factors strongly influence the conversion process. Among the interaction terms, only the interaction between time and temperature was significant with a *p* value of 0.0369, while the interactions between time and DES dosage and between temperature and DES dosage were not significant. For the quadratic effects, the square of reaction time showed clear curvature with a *p* value of 0.0005, whereas the square of DES dosage was marginally significant and the square of temperature was not significant. The lack-of-fit test showed a *p* value of 0.2614, demonstrating that the model adequately fits the experimental data. The coefficient of determination *R* squared was 0.9722 and the adjusted *R* squared was 0.9364, both indicating excellent model performance, while the predicted *R* squared of 0.7173 suggests acceptable predictive capability. The adequate precision value of 16.87 also reflects a strong signal-to-noise ratio. Overall, these results confirm that the regression model is reliable and suitable for optimizing 5-HMF production from jackfruit rind.

**Table 4:** Analysis of variance (ANOVA) results

Source	Sum of squares	df	Mean square	*F*-Value	*p*-Value
Model	4417.87	9	490.87	27.19	0.0001
*A* – Reaction time	2342.36	1	2342.36	129.73	<0.0001
*B* – Reaction temperature	578.17	1	578.17	32.02	0.0008
*C* – DES dosage	613.9	1	613.9	34	0.0006
*AB*	119.36	1	119.36	6.61	0.0369
*AC*	16.16	1	16.16	0.8951	0.3756
*BC*	24.9	1	24.9	1.38	0.2787
*A* ^2^	652.14	1	652.14	36.12	0.0005
*B* ^2^	0.0466	1	0.0466	0.0026	0.9609
*C* ^2^	93.69	1	93.69	5.19	0.0568
Residual	126.39	7	18.06		
Lack of fit	75.29	3	25.1	1.96	0.2614
Pure error	51.1	4	12.77		
Cor. total	4544.26	16			

**Fig. 3:**
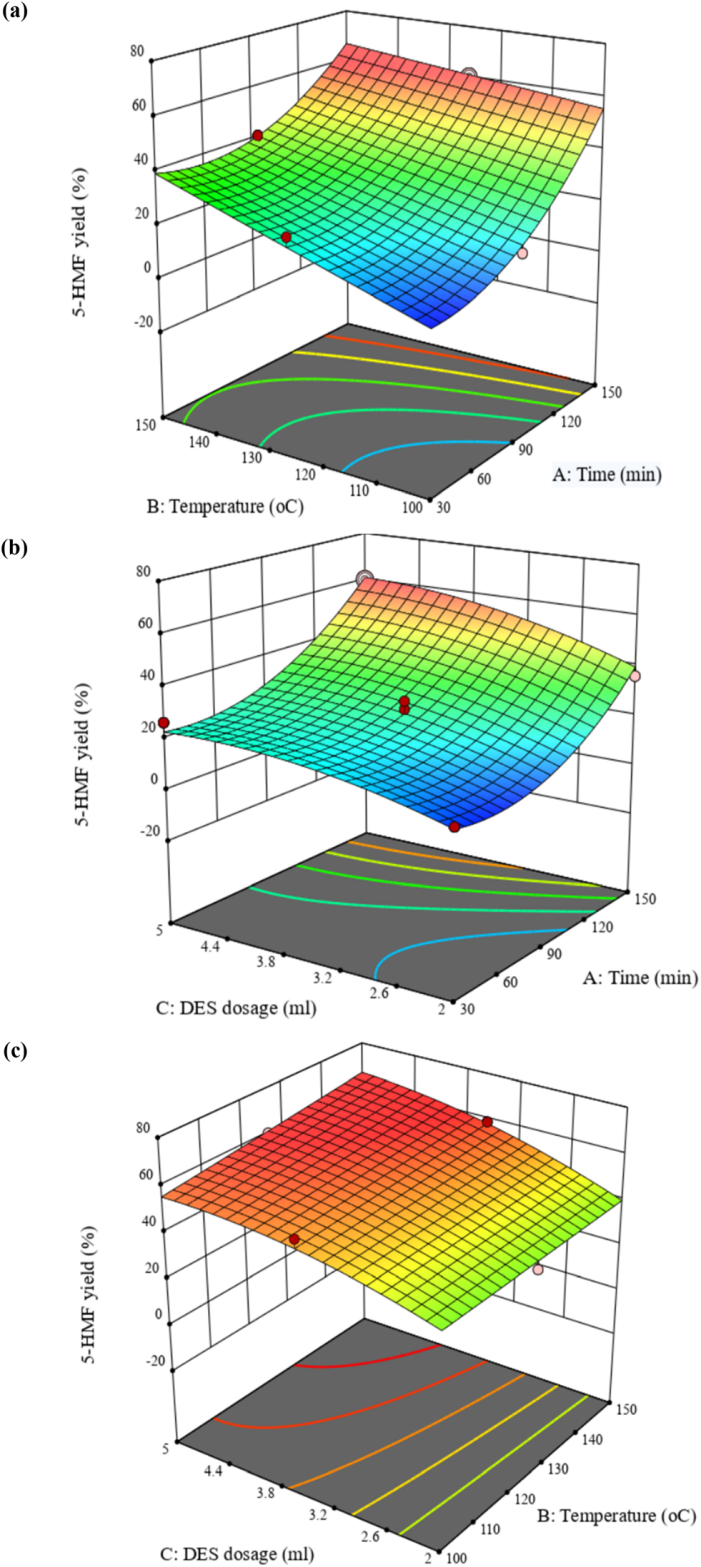
Response surface (3-D) and contour (2-D) plots about the interaction of factors in the 5-HMF yield: (a) reaction temperature and reaction time; (b) DES dosage and reaction time; (c) DES dosage and reaction temperature.

As shown in Fig. 3, the model was used to produce three-dimensional response surfaces and two-dimensional contour plots to demonstrate how the variables interacted with each other and to estimate the optimal levels for the maximum response of each variable.

The plots showed an intricate relationship between the reaction parameters – time, temperature, and DES dosage – and the result of 5-HMF yield. 5-HMF formation increases markedly with longer reaction time and higher temperature. At 150 min and 125 °C, the yield reached the highest value of 60.18%, while the same temperature at 30 min produced only 10.20%. At lower temperatures such as 100 °C, yields remained modest, ranging from 5.89 to 30.38% depending on DES dosage and time. Increasing DES dosage from 2 to 3.5 mL generally improved performance, with yields rising from 21.02 to 31.04% at 150 °C and from 15.67 to 23.43% at 100 °C. However, at the most severe conditions of 150 °C, further increases in DES dosage did not consistently enhance yield, suggesting possible onset of 5-HMF degradation.

Based on eqn (2) and using the software's numerical optimization function, the maximum predicted 5-HMF yield was 66.42% for the optimal conditions of a 150 min reaction time, 5 mL of DES, and 135 °C reaction temperature. Three experiments were conducted under optimal conditions to verify the prediction, and a 5-HMF yield of approximately 63.42% ± 1.46% was obtained. This demonstrated that the experimental data were consistent with the model.

### 3.5. DFT calculation for mechanisms proposal

DFT calculations were carried out to clarify the molecular-level transformation of glucose within the HBetCl:AlCl_3_ DES. Two principal routes were investigated. The first involves direct dehydration of glucose to 5-hydroxymethylfurfural under Brønsted acid catalysis. The second follows a cooperative Brønsted–Lewis acid pathway in which glucose first isomerizes to fructose and then undergoes sequential dehydration to form 5-hydroxymethylfurfural. These computational results make it possible to identify the energetically preferred route and the specific roles of the acidic sites in the deep eutectic medium.

**Fig. 4:**
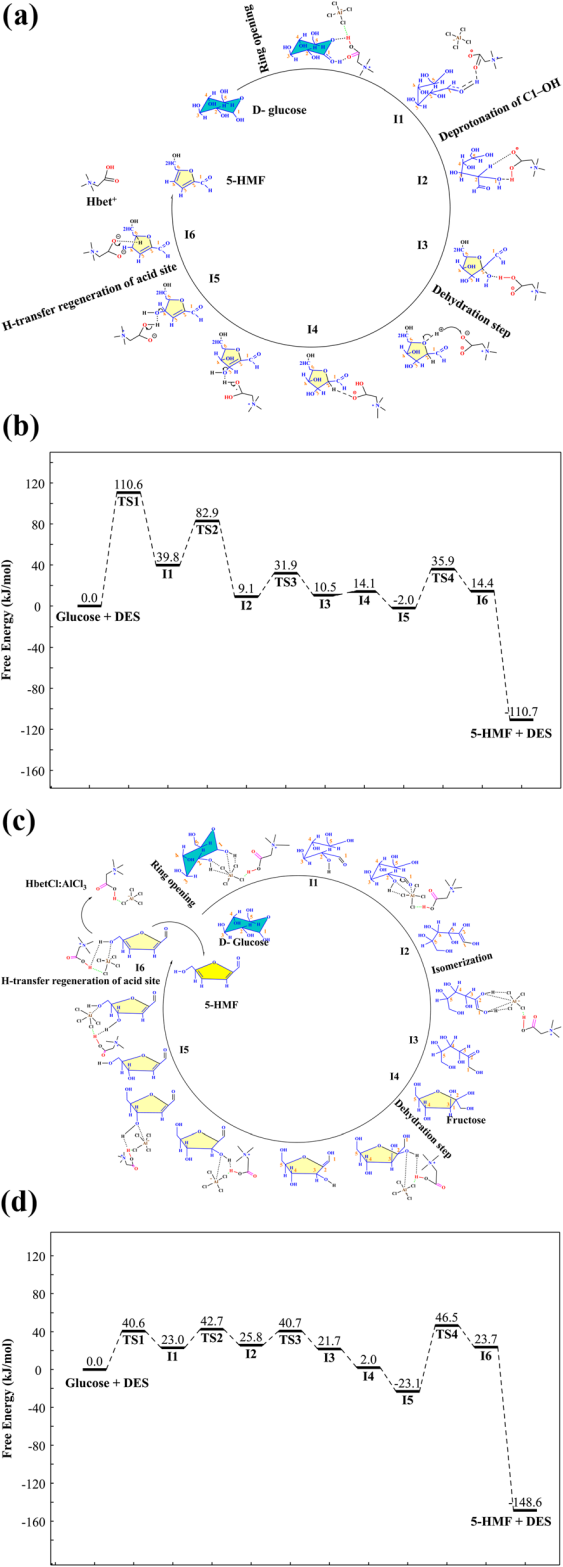
(a) The process and (b) DFT calculation for direct dehydration of glucose to 5-HMF under Brønsted acid catalysis; (c) the process and (d) DFT calculation for dehydration of glucose to 5-HMF under Brønsted–Lewis acid catalysis.

The conversion of d-glucose to 5-HMF directly without isomerization to fructose is facilitated by the Brønsted acid site of HBetCl:AlCl_3_ DES through a highly efficient, metal-free pathway. As shown in Fig. 4 (a) and (b), the process begins with protonation of the C1–OH group at intermediate I1, followed by ring opening of the pyranose structure *via* transition state TS1 with an energy barrier of 110.6 kJ mol^−1^, forming an open-chain intermediate. Subsequent H-transfer regenerates the acid site at I5 and enables sequential deprotonation of the C1–OH at I2 and dehydration steps at I3 and I4, with the rate-determining TS3 at 31.9 kJ mol^−1^ involving C2–OH elimination. Final dehydration at the C3 position through TS4 with a barrier of 35.9 kJ mol^−1^ forms 5-HMF at I6, achieving an overall energy drop from 0.0 kJ mol^−1^ for glucose + DES to −110.7 kJ mol^−1^ for 5-HMF + DES.

The conversion of glucose to 5-HMF using both Brønsted and Lewis acid sites of HBetCl:AlCl_3_ DES follows a stepwise mechanism that includes isomerization to fructose. As shown in Fig. 4 (c) and (d), the process starts with protonation and ring opening of glucose through TS1 at 40.6 kJ mol^−1^, followed by hydrogen transfer to regenerate the acid site at I5. Glucose then isomerizes to fructose *via* intermediates I2 and I3, passing transition states TS2 at 42.7 kJ mol^−1^ and TS3 at 40.7 kJ mol^−1^. From fructose at I4, dehydration removes water from C2 and C3 positions through TS4 at 46.5 kJ mol^−1^, forming 5-HMF at I6. The reaction is highly exothermic, dropping from 0.0 kJ mol^−1^ for glucose + DES to −148.6 kJ mol^−1^ for 5-HMF + DES, reflecting strong thermodynamic favorability and efficient dual-acid catalysis.

Compared to the direct Brønsted-only path, the dual-acid route *via* fructose is more efficient. The direct path faces a high initial barrier of 110.6 kJ mol^−1^ for ring opening and a rate-determining step of 31.9 kJ mol^−1^, with total energy release of 110.7 kJ mol^−1^. In contrast, the fructose pathway reduces the highest barrier to 46.5 kJ mol^−1^ and increases energy release to 148.6 kJ mol^−1^, making it faster and more stable. The result of substantial reduction in activation energy confirm that, in this DES system, glucose predominantly transforms into 5-HMF through fructose, reflecting the synergistic role of Lewis acid coordination in promoting isomerization over direct dehydration.

**Fig. 5:**
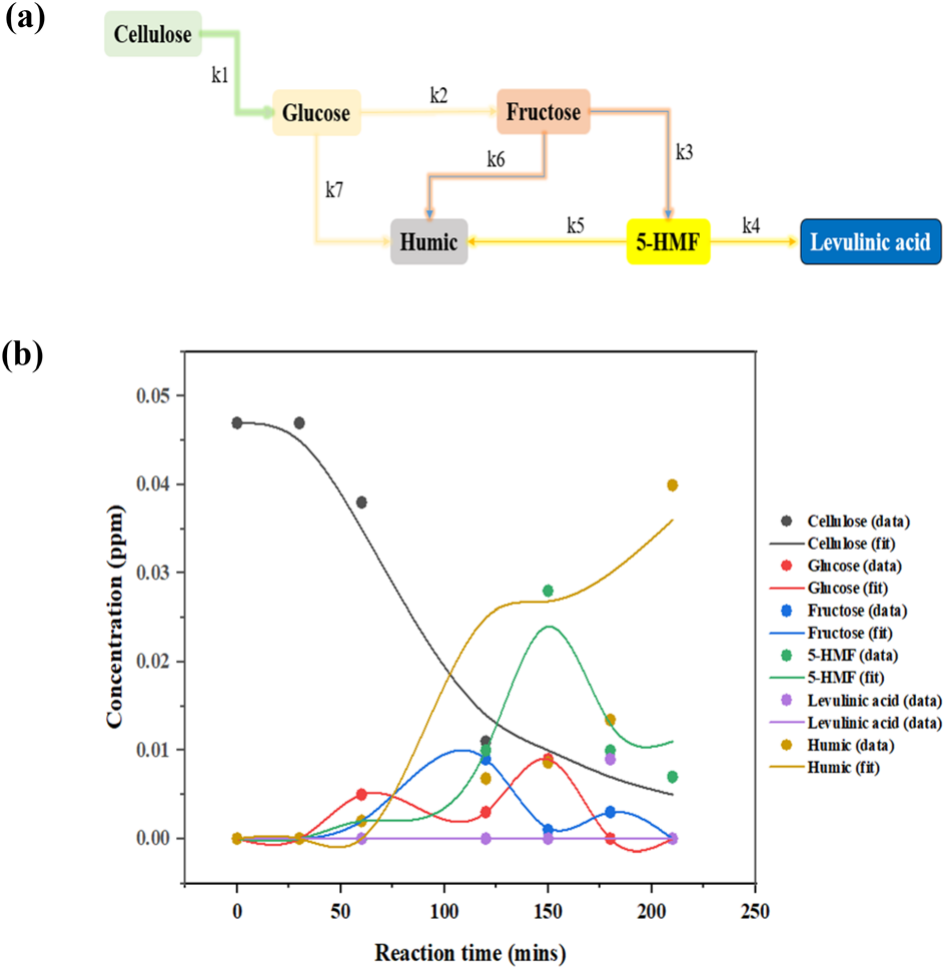
(a) A simplified pathway of the overarching kinetic model; (b) the simulated curves *vs.* the experimental data.

Guided by these computational insights, a simplified kinetic scheme assuming pseudo-first-order behavior in each elementary step was proposed and validated experimentally. In this study, we operated under the assumption of a well-mixed, isothermal reactor throughout the reaction duration, and irreversible reactions. Utilizing a monophasic model grounded inhomogeneous, the reaction first facilitated the decomposition of cellulose into glucose, which then isomerized to fructose and underwent dehydration to yield 5-HMF. The resultant 5-HMF was further subjected to rehydration, leading to the production of levulinic acid. It is pertinent to note that glucose, fructose, and 5-HMF are reactants with the theoretical capacity to generate by-products, such as humic substances. A simplified depiction of the overarching kinetic model employed in this study is presented in Fig. 5(a).

The kinetic parameter estimation is based on the following set of eqn (3)–(7):3
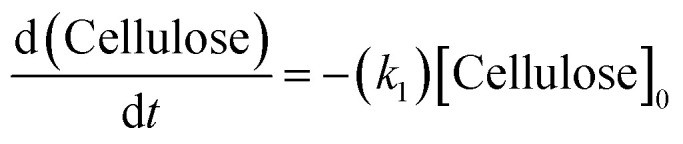
4
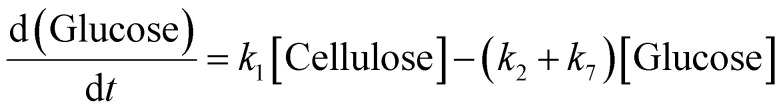
5
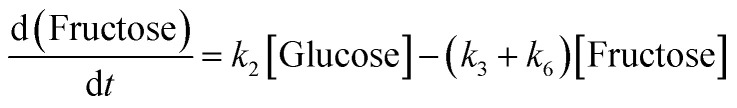
6
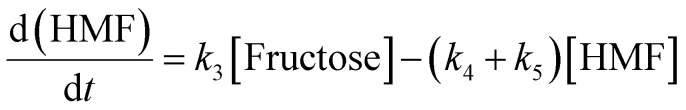
7
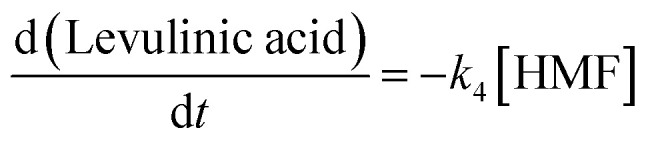
[Cellulose]_0_, [Cellulose], [Glucose], [Fructose], [HMF], and [Levulinic acid] refer to initial concentration of glucan, the concentration of cellulose, glucose, fructose, 5-HMF, and levulinic acid at the time “*t*”, respectively. Meanwhile, *k*_1_, *k*_2_, *k*_3_, *k*_4_, *k*_5_, *k*_6_ and *k*_7_ are the kinetic constants of glucan decomposition to glucose, glucose to 5-HMF, 5-HMF to levulinic acid, glucan to by-products, glucose to by-products, and 5-HMF to by-products. The reaction rate constants for both kinetic studies were computed by using the fourth-order Runge–Kutta (RK4) method with the help of Matlab software.

The activation energy (*E*_a_) and exponential factor (*A*) were obtained by applying the following Arrhenius equation (eqn (8)) to the experimental data:8
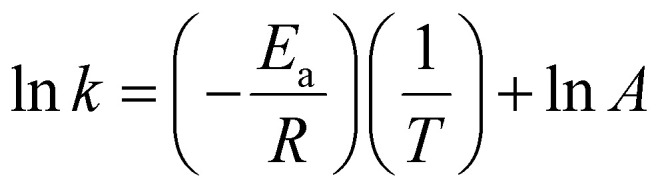


To evaluate the reliability of the kinetic model and the obtained rate constants, a series of validation experiments were conducted by varying the reaction time from 30 minutes to 210 minutes 125 °C. As shown in Fig. 5(b), the simulated curves closely match the experimental data, confirming that the fitted kinetic parameters accurately capture the temperature-dependent behavior of cellulose conversion and product formation.

The temperature dependence of the apparent rate constants was analyzed over the range 100–150 °C. As expected, all seven rate constants increase with temperature, following the Arrhenius relationship with excellent linearity (*R*^2^ ≥ 0.80). The derived activation energies are summarized in Table 5.

**Table 5:** Rate constants and derived activation energies

Re. step (min^−1^)	Description	150 °C	125 °C	100 °C	*E*_a_ (kJ mol^−1^)	*R* ^2^
*k*_1_	Cellulose to glucose	0.0932	0.0499	0.0385	22.97	0.93
*k*_2_	Glucose to fructose	0.0716	0.0413	0.0289	23.63	0.97
*k*_3_	Fructose to HMF	0.1085	0.0749	0.0568	16.90	0.99
*k*_4_	5-HMF to Le. acid	0.0389	0.0185	0.0010	97.21	0.92
*k*_5_	5-HMF to humic	0.0894	0.0398	0.0113	54.55	0.99
*k*_6_	Fructose to humic	0.0413	0.0284	0.0099	37.88	0.95
*k*_7_	Glucose to humic	0.0199	0.0169	0.0026	54.46	0.84

The dehydration of fructose to HMF (*k*_3_) exhibits the lowest activation energy (16.90 kJ mol^−1^), making it the least temperature-sensitive step in the desired pathway. In contrast, both two major degradation routes of HMF: rehydration to levulinic acid (*k*_4_, *E*_a_ = 97.21 kJ mol^−1^) and polymerization to humic substances (*k*_5_, *E*_a_ = 54.55 kJ mol^−1^), possess significantly higher activation barriers. Similarly, the direct degradation of glucose to humic substances (*k*_7_) shows the high *E*_a_ (54.46 kJ mol^−1^) in the entire network. This activation-energy profile provides a clear kinetic rationale for maximizing 5-HMF selectivity. Although high temperature (≥150 °C) is required for rapid cellulose hydrolysis (*k*_1_) and acceptable overall conversion rates, the undesired high-*E*_a_ degradation pathways (particularly *k*_4_, *k*_5_, and *k*_7_) are occurred concurrently and strongly. Consequently, a two-stage temperature strategy, initial rapid heating to 140–150 °C to achieve fast cellulose depolymerization and HMF formation, followed by rapid cooling to ≤120 °C, is predicted to dramatically improve 5-HMF yield by kinetically quenching the degradation reactions while preserving sufficient rate for the low-*E*_a_*k*_3_ step.

### 3.6. DES reusability

**Fig. 6:**
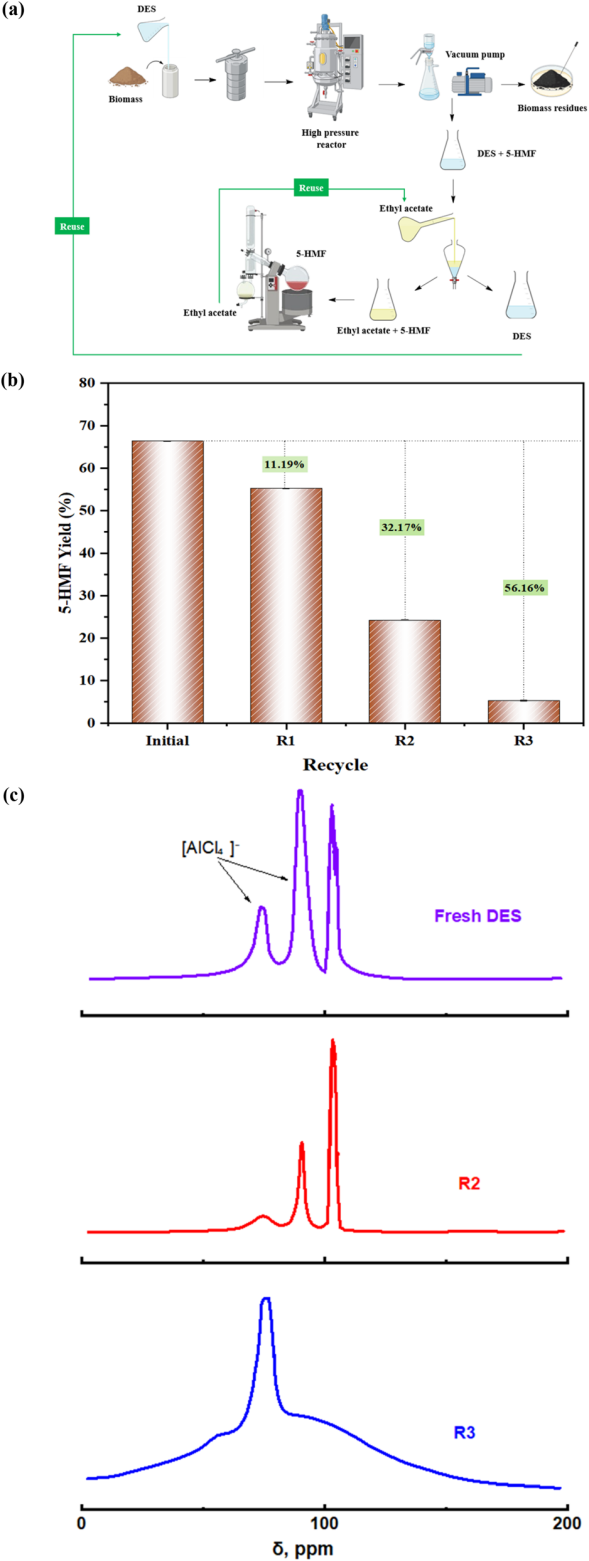
(a) The process of the experiment and reuse; (b) the reusability of DES with reaction condition: 200 mg of jackfruit rinds as the reaction substrate, 5 mL of DES, 10% of mL DI water, and 135 °C heating in high pressure reactor in 150 min; (c) ^27^Al NMR spectra of fresh DES, second run, and four run.

DES and ethyl acetate are fully recycled as displayed in Fig. 6(a): after extraction of 5-HMF into ethyl acetate, the solvent is recovered by distillation and reused; the aqueous DES phase is concentrated under vacuum and directly charged to the next reaction cycle together with fresh biomass.

The reusability of the DES was examined over four consecutive reaction cycles under identical conditions, as shown in Fig. 6(b). The fresh DES produced an excellent 5-HMF yield of 66.42%. In the first recycle, the yield remained relatively high at 55.23%, corresponding to 11.19% loss of the initial activity, which indicates good short-term stability of the catalytic system. However, subsequent cycles exhibited a pronounced decline in performance, with yields dropping to 24.25% in the second recycle and only 5.26% in the third with 32.17% loss and 56.16% loss, respectively, of the initial activity. Although this trend might initially appear to reflect simple catalyst deactivation, the observed loss of activity is attributed to progressive contamination and alteration of the DES.

To investigate the structural stability of the DES upon recycling, ^27^Al NMR spectra of fresh, second run, and fourth run DES were displayed in Fig. 6(c). The fresh DES exhibits characteristic tetrahedral chloroaluminate signals at around 90–100 ppm corresponding to [AlCl_4_]^−^ species.^40^ After the *R*^2^, these signals remain largely unchanged, indicating preservation of the Lewis acidic species. However, after the R3, peak broadening and partial signal merging were observed, suggesting some coordination changes and structural rearrangement of chloroaluminate species during extended reuse.

### 3.7. Other biomass conversion and the comparisons

To expand the horizon of fruit waste utilization, various fruit wastes, including pineapple peel, banana peel, and durian rind, were applied the optimal reaction condition finding and their comparisons to previous studies, which used DES for 5-HMF synthesis. The results in Table 6 clearly demonstrate the superior performance and broad applicability of the HBetCl:AlCl_3_ DES system for direct, pretreatment-free conversion of untreated lignocellulosic biomass to 5-HMF.

**Table 6:** Comparison of 5-HMF yields from using HBetCl:AlCl_3_ DES (this study) *versus* literature systems

Biomass source	Catalytic/solvent system	Temp. (°C)	Time (min)	5-HMF yield (%)
Pineapple stem^33^	ChCl:Lac/DMSO	150	120	40.98
Corn stover^41^	HCl	140	120	42.00
Corn stover^41^	HCl	140	120	48.00
d-Glucose^35^	ChCl:MA/DMSO	150	30	85.60
d-Fructose^42^	ChCl:Fruc/MeCN/H_3_SW_12_O_40_	80	15	70.00
Jackfruit rind (this study)	HBetCl:AlCl_3_	135	150	66.42
Pineapple peel (this study)	HBetCl:AlCl_3_	135	150	62.18
Banana peel (this study)	HBetCl:AlCl_3_	135	150	52.36
Durian rind (this study)	HBetCl:AlCl_3_	135	150	53.12

Among real agro-wastes, jackfruit rind achieves the highest yield at 66.42%, closely followed by pineapple peel at 62.18%, banana peel at 52.36%, and durian rind at 53.12%. These trends align with biomass recalcitrance: fruit-derived wastes with particularly low-lignin peels/rinds outperform grassy residues due to greater carbohydrate accessibility and reduced humin formation. In the compared observation, the yields from jackfruit rind and pineapple peel approach are superior with pineapple stem at 40.98% by using ChCl:Lac/DMSO system, and corn stover by using ionic liquid [EMIM]Cl based system, ranging from 42 to 48%. Furthermore, these results remain competitive when compared to those from pure d-glucose at 85.6% and d-fructose at 70%, despite the absence of organic extractant phase, mineral acids, or pretreatment in this monophasic system. The consistently high performance across diverse tropical fruit wastes highlights the HBetCl:AlCl_3_ DES's exceptional efficiency for carbohydrate-rich, low-lignin agro-residues for sustainable biorefinery applications in fruit-processing regions.

### 3.8. Life cycle assessment

#### 3.8.1. LCA results

**Fig. 7:**
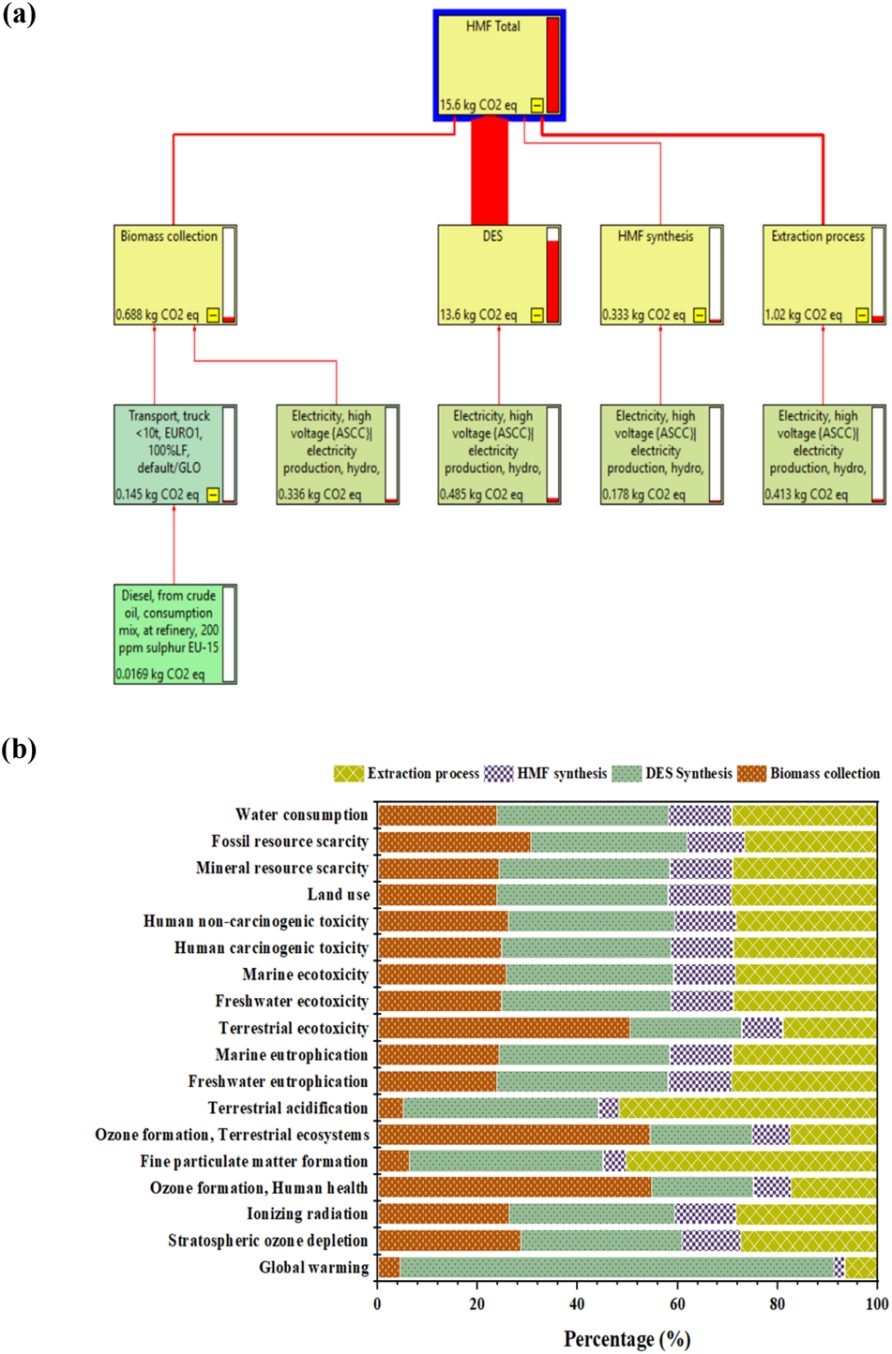
Life cycle assessment results (a) CO_2_ eq. contributions; (b) ReCiPe 2016 midpoint categories.

The life cycle assessment indicates that the HBetCl:AlCl_3_ deep eutectic solvent (DES) process for direct 5-HMF production from untreated jackfruit rind exhibits a total global warming potential of 15.6 kg CO_2_ eq. within the defined cradle-to-gate system boundary, as shown in Fig. 7(a).

Contribution analysis reveals that DES synthesis is the dominant source of greenhouse gas emissions, accounting for 13.6 kg CO_2_ eq., corresponding to approximately 87% of the total impact. This high contribution reflects the upstream energy demand associated with DES component preparation and electricity consumption. In contrast, the extraction process contributes 1.02 kg CO_2_ eq. (6.5%), while biomass collection contributes 0.688 kg CO_2_ eq. (4.4%). The 5-HMF synthesis stage shows the lowest contribution at 0.333 kg CO_2_ eq., representing approximately 2.1% of the total, confirming that the core reaction step imposes a minimal climate burden due to its high efficiency and selectivity.

The ReCiPe 2016 midpoint analysis reveals distinct stage-specific environmental hotspots across impact categories, as shown in Fig. 7(b). DES synthesis dominates global warming, contributing 86.92% of the total impact, and similarly leads stratospheric ozone depletion, ionizing radiation, and several toxicity-related categories, reflecting the upstream energy demand and material intensity associated with DES component production. In contrast, biomass collection strongly influences air-quality-related impacts, accounting for 54.83% of ozone formation for human health and 54.56% for terrestrial ecosystems, primarily due to transport-related NO_*x*_ emissions. The extraction process emerges as a major contributor to fine particulate matter formation with 50.34%, terrestrial acidification with 51.83%, and water consumption with 29.26%, driven by electricity use during solvent recovery and distillation.

Across all midpoint categories, the 5-HMF synthesis stage consistently shows the lowest contribution, generally remaining below 13%, confirming that the core conversion step does not represent an environmental hotspot. Eutrophication-related impacts are more evenly distributed among DES synthesis, extraction, and biomass collection, while resource-related categories indicate notable contributions from DES synthesis to mineral resource scarcity with 34.10% and from biomass collection to fossil resource scarcity with 30.70%. Overall, the results indicate that the environmental performance of the system is governed primarily by upstream DES preparation and downstream extraction, whereas the high efficiency and selectivity of the HMF synthesis step minimize its relative contribution, highlighting DES recyclability and energy optimization as key levers for further impact reduction.

As shown in Table 7, the GWP of the present HBetCl:AlCl_3_ DES-based process is 15.6 kg CO_2_ eq. per kg HMF. Although this value is higher than some reported sugar-based routes, such differences mainly arise from variations in system boundaries and allocation assumptions. Many literature studies reporting lower GWP values rely on refined sugar feedstocks and apply simplified boundaries in which the synthesis of catalyst and solvent components is excluded or assumed to be impact-free, resulting in optimistic estimates. By comparison, the present assessment accounts for all material and energy inputs, including the synthesis of DES components, make-up losses, and process energy consumption, in a consistent cradle-to-gate framework. The comprehensive analysis has demonstrated the technical feasibility of producing 5-HMF from unprocessed agricultural waste employing the recyclable DES system.

**Table 7:** Comparison of cradle-to-gate GWP for 5-HMF production

Feedstock	Process/solvent/catalyst	GWP (kg CO_2_ eq. per kg HMF)
Jackfruit rind (this study)	HBetCl:AlCl_3_ DES	15.60 kg
Maize based HFCS^43^	Sulfuric acid and water	0.46–2.24 kg
Sago pith waste^44^	The DMSO–water system	2.10–9.70 kg
Fructose^45^	H_2_SO_4_, TsOH, and FeCl_3_ grafted biochar catalyst in i-PrOH/water	477 kg

#### 3.8.2. Sensitivity analysis

A sensitivity analysis was conducted on two key parameters, DES reuse and renewable energy substitution, under three scenarios to evaluate their potential for reducing CO_2_ emissions. Three scenarios were examined: (i) DES reuse across multiple cycles with DES synthesis treated as a one-time emission, (ii) renewable electricity substitution through partial replacement of grid electricity, and (iii) a combined strategy integrating both DES reuse and renewable electricity substitution.

**Fig. 8:**
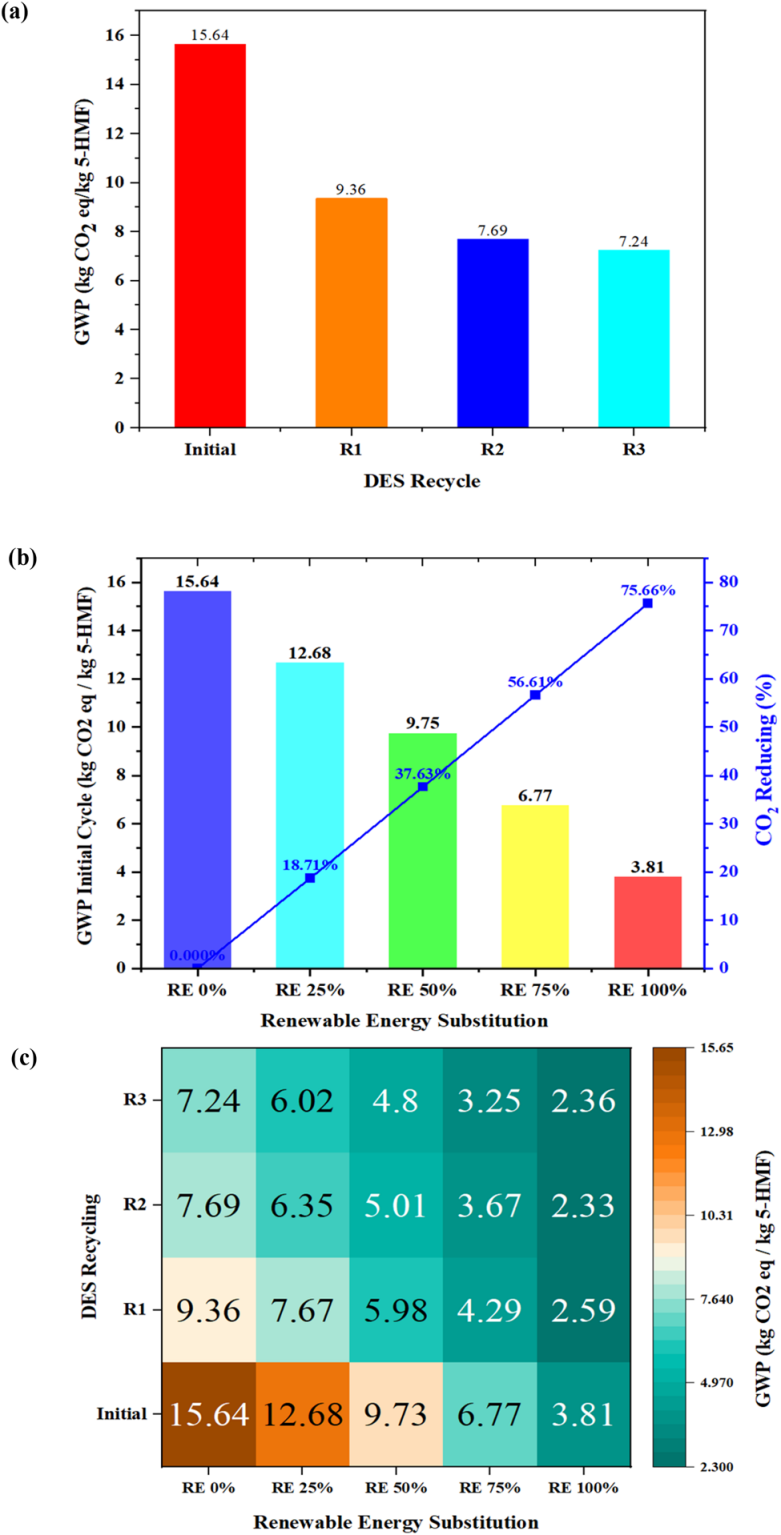
Sensitivity analysis; (a) the DES is reused; (b) renewable electricity substitution; (c) the combined effects of DES reuse and renewable electricity substitution.

The sensitivity analysis results demonstrate that both DES reuse and renewable electricity substitution significantly influence the environmental performance of the process. As shown in Fig. 8(a), the base-case scenario exhibits a GWP of 15.64 kg CO_2_ eq. per kg HMF. When the DES is reused across successive reaction cycles, the GWP decreases substantially due to the amortization of the DES synthesis burden. Specifically, the GWP decreases to 9.36 kg CO_2_ eq. per kg HMF in the first recycle (R1) and further declines to 7.69 kg CO_2_ eq. per kg HMF in *R*^2^, reaching 7.24 kg CO_2_ eq. per kg HMF in R3. This corresponds to an overall reduction of approximately 54% relative to the initial case, indicating that the environmental impact of DES synthesis is a major hotspot and can be significantly mitigated through catalyst reuse.

Renewable electricity substitution also exhibits a strong influence on process emissions. As illustrated in Fig. 8(b), replacing grid electricity with hydropower progressively reduces the GWP from 15.64 kg CO_2_ eq. per kg HMF (0% renewable electricity) to 12.68, 9.75, 6.77, and 3.81 kg CO_2_ eq. per kg HMF at 25%, 50%, 75%, and 100% renewable electricity, respectively. Under full renewable electricity supply, the total GWP decreases by approximately 75.70%, highlighting the significant contribution of electricity consumption to the overall environmental footprint.

The combined effects of DES reuse and renewable electricity substitution are summarized in Fig. 8(c). When both strategies are applied simultaneously, the GWP can be further reduced to 2.36 kg CO_2_ eq. per kg HMF under the most favorable condition at R3 with 100% renewable electricity. This represents an overall reduction of approximately 85% compared with the base case, demonstrating that process optimization and energy decarbonization together provide the most effective pathway for improving environmental competitiveness.

In sum, the sensitivity analysis indicates that DES reuse primarily reduces the embodied emissions associated with catalyst synthesis, while renewable electricity substitution mitigates operational energy emissions. The synergistic implementation of these strategies substantially improves the environmental performance of the process and suggests that sustainable electricity supply and improved catalyst stability will be key factors for achieving competitive low-carbon production of 5-HMF from biomass.

## 4. Conclusion

This study presents an efficient, catalyst-free conversion of biomass, a low-value, lignin-poor agro-waste, into 5-HMF using a bifunctional Brønsted–Lewis acidic DES HBetCl:AlCl_3_. Under optimized conditions: *T* = 135 °C, *t* = 150 min, water content = 10 vol%, HBetCl : AlCl_3_ ratio = 1 : 0.5, a high yield of 66.42 ± 0.15 mol% of 5-HMF was achieved directly from jackfruit rind without any treatment. Pyridine-FTIR and TGA analysis jointly proved the adjustable dual Brønsted–Lewis acidity and high thermal stability of the DES (>220 °C onset), and the RSM optimization allowed for accurate optimization of reaction conditions. Notably, DFT calculations and kinetic analysis showed that the high performance is due to the synergistic effect of dual Brønsted–Lewis acid catalysis: the presence of Lewis acidic chloroaluminate significantly reduces the energy barrier for glucose-to-fructose isomerization, making the reaction proceed mainly through the fructose dehydration pathway with the highest barrier of 46 kJ mol^−1^ compared to >110 kJ mol^−1^ for direct glucose dehydration. Moreover, LCA analysis shows that the process has positive environmental impacts, with a global warming potential of 15.6 kg CO_2_ eq. per kg of 5-HMF produced. Although the proposed system has the advantage of not using mineral acids, toxic solvents, or biomass pretreatment, there are a few limitations to the proposed method. Among these, the recyclability of the DES was found to have a considerable decline in the activity of the catalyst after a series of cycles, which must be addressed by developing an effective regeneration protocol. Additionally, a techno-economic analysis of the proposed method must be carried out to assess its viability on a larger scale. Overall, the HBetCl:AlCl_3_ DES system has tremendous potential to utilize the less utilized lignocellulosic waste to produce the valuable chemical 5-HMF, which can be used as a precursor to more eco-friendly bio-refinery technologies.

## Author contributions

Q. T. Huynh: writing – original draft; formal analysis; visualization; methodology; investigation. U. Jaitham: formal analysis, methodology, visualization; H. N. Tran: writing – review and editing, formal analysis, methodology. All authors have read and agreed to the published version of the manuscript. All authors have read and agreed to the published version of the manuscript.

## Conflicts of interest

The authors declare no competing interests.

## Supplementary Material

RA-016-D6RA01120A-s001

## Data Availability

The data supporting the findings of this study are available within the article and its supplementary information (SI). Additional data, including raw experimental measurements, computational inputs and outputs, and life cycle inventory data, are available from the corresponding author upon reasonable request. Supplementary information detailed biomass compositional analysis, MATLAB code for kinetic analysis, and life cycle assessment modeling and methodology. See DOI: https://doi.org/10.1039/d6ra01120a.
