# Supplementary material for: Direct conversion of underutilized tropical fruit wastes to 5-hydroxymethylfurfural using a strongly acidic deep eutectic solvent: mechanistic study, renewable extraction, and life cycle assessment
Source: RSC Adv. 2026 Mar 24;16(18):16491–504. doi: 10.1039/d6ra01120a (PMC13011918; doi:10.1039/d6ra01120a)
Supplement: RA-016-D6RA01120A-s001 [file RA-016-D6RA01120A-s001.pdf]

Supplementary Information (SI) for RSC Advances.
This journal is © The Royal Society of Chemistry 2026

Supplementary Information

Direct conversion of under-utilized tropical fruit wastes to 5-hydroxymethylfurfural using a superacidic deep eutectic solvent: Mechanistic study, renewable extraction, and life cycle assessment

*Quang Tam Huynh [1, *], Udomsap Jaitham [2], Hai Nguyen Tran [1, 3]*

[1]Institute of Fundamental and Applied Sciences, Duy Tan University, Ho Chi Minh City 70000, Viet Nam.

[2]School of Health Sciences Research, Research Institute for Health Sciences, Chiang Mai University, Chiang Mai 50200, Thailand.

[3]Faculty of Environment and Chemical Engineering, Duy Tan University, Da Nang 55000, Viet Nam.

[4]Institute of Environmental Engineering, National Sun Yat-Sen University, Kaohsiung 804, Taiwan.

[5]Net Zero Emissions and Resource Recycling Technology Research Center, National Sun Yat-Sen University, Kaohsiung 804, Taiwan.

[6]Department of Public Health, College of Health Sciences, Kaohsiung Medical University, Kaohsiung 807, Taiwan.

[7]Center for Emerging Contaminants Research, National Sun Yat-Sen University, Kaohsiung 804, Taiwan.

[8]Environment, Occupational Health Sciences and Non-Communicable Disease Center of Excellence, Re-search Institute for Health Sciences, Chiang Mai University, Chiang Mai 50200, Thailand.

*Corresponding author at: Institute of Fundamental and Applied Sciences, Duy Tan University, Ho Chi Minh City 70000, Viet Nam.

E-mail address: huynhqtam85@gmail.com *(Q.T. Huynh)*.

I. Component analysis

1. Lignocellulosic biomass

Following the National Renewable Energy Laboratory (NREL) guidelines.

Acid treatment was begun with 300 mg of biomass mixing with 3 mL of 72% sulfuric acid in a 100 mL high-pressure tube. This mixture was incubated in a water bath at 30 °C for one hour with continuous agitation. Following the incubation, 84 mL of distilled water was added to dilute the mixture, which was then autoclaved at 120 °C for one hour. The hydrolyzed sample was filtered under vacuum, and the remaining acid-insoluble fraction was thoroughly rinsed with deionized water to remove residual acid.

Lignin and carbohydrate content were quantified using a combination of spectroscopic and chromatographic methods. Acid-soluble lignin (ASL) was measured via UV spectroscopy at 205 nm using a Shimadzu UV-1700. The acid-insoluble residue (AIR) was oven-dried at 105 °C overnight and subsequently incinerated in a muffle furnace at 575 °C to determine acid-insoluble ash (AIA). Klason lignin (KL) was calculated by subtracting AIA from AIR, while total lignin (TL) content was the sum of ASL and KL. For cellulose and hemicellulose analysis, 20 mL of the filtrate was neutralized to pH 5–6 using calcium carbonate. This solution was analyzed using HPLC (Hitachi CM5000, Japan) equipped with a BP-8000 Ca column (300 × 7.8 mm) and an RI detector. The mobile phase consisted of purified water at a flow rate of 0.6 mL/min, with the column maintained at 85 °C over a 20-minute run time.

Using the following formula, the percentage of cellulose or hemicellulose was determined by equation 1:

$$\% \, Cellulose\ or\ Hemicellulose = \frac{\text{HPLC concentration}\left(\frac{\text{mg}}{\text{l}}\right) * \ Dilution\ (\text{l}) \ * \ \text{AC}}{ODW_{sample}\ (\text{mg})} \text{ x } 100 \tag{1}$$

Where:

- AC: Using an anhydrous correction of 0.88 for C-5 sugars (Cellulose) and a correction of 0.90 for C-6 sugars (Hemicellulose).

- ODW_{sample}: Weight of sample.

- Dilution: Volume of diluting solvents.

2. Glucose and Fructose

The obtained glucose and fructose concentrations were determined by the HPLC (Hitachi CM5000, Japan) equipped with a BP-8000 Ca column (300 × 7.8 mm) and an RI detector. The mobile phase consisted of purified water at a flow rate of 0.6 mL/min, with the column maintained at 85 °C over a 20-minute run time. Quantitative analyses were conducted using the external standard method with standard solutions were prepared at concentrations of 25 ppm, 50 ppm, 75 ppm, 100 ppm, and 150 ppm, which were then used to construct a calibration curve as depicted in Figure S1

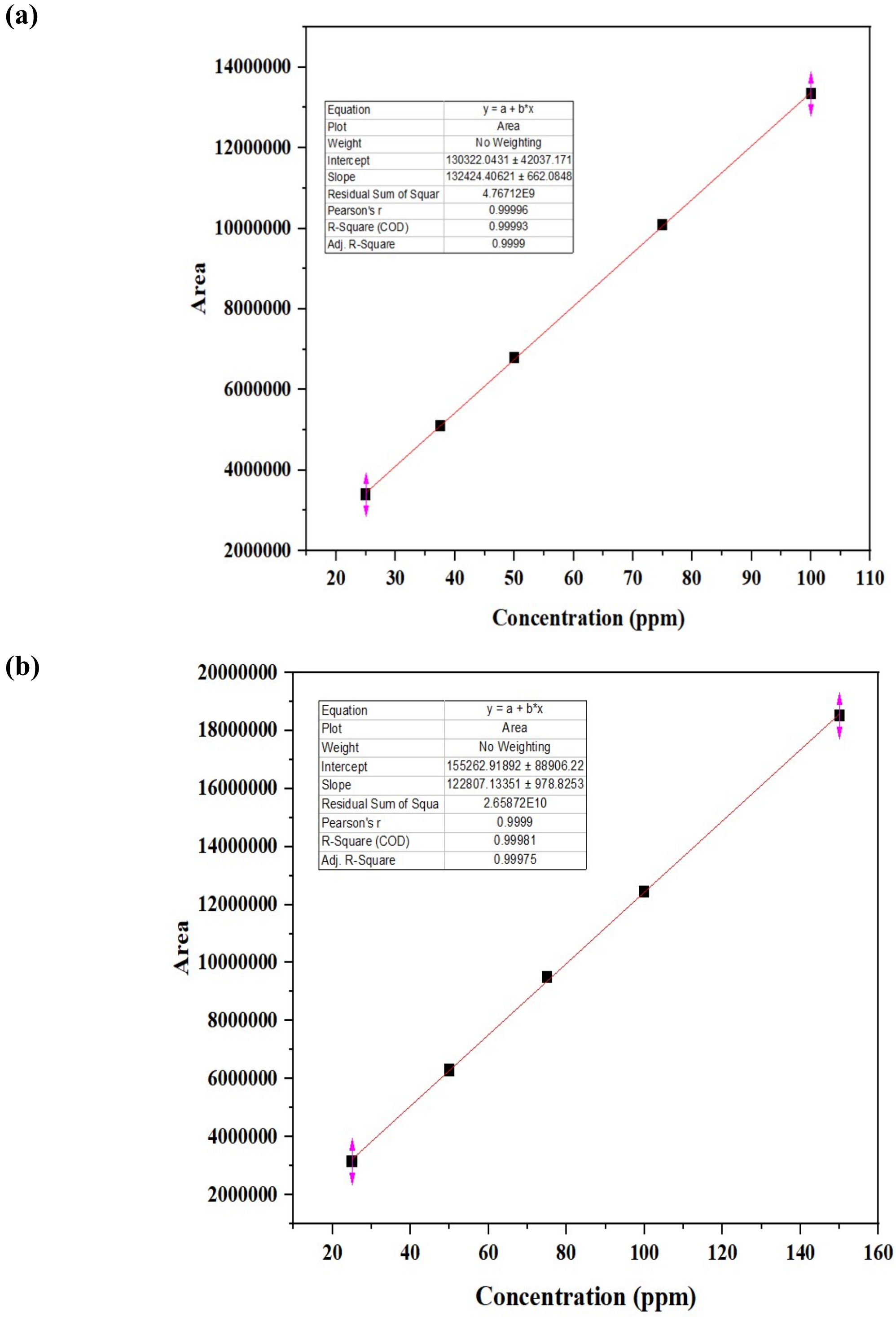

Figure S1: Calibration curve of (a)- Glucose; (b)- Fructose

II. MATLAB code for mechanism model

```
lear; clc; close all;
%% Experimental data
data = [
    0    0.047  0.000  0.000  0.000  0.000  0.000   % t=0
    30    0.047  0.000  0.000  0.000  0.000  0.000   % t=30
    60    0.038  0.005  0.002  0.000  0.000  0.002   % t=60
   120    0.011  0.003  0.009  0.010  0.000  0.006   % t=120
   150    0.000  0.009  0.001  0.028  0.000  0.009   % t=150
   180    0.000  0.000  0.003  0.010  0.009  0.013   % t=180
   210    0.000  0.000  0.000  0.007  0.000  0.040];% t=210
t_exp = data(:,1);
y_exp = data(:,2:7);       % [Cellulose, Glucose, Fructose, HMF, LA, By-products]
C0 = 0.047;
y0 = [C0  0  0  0  0  0];
%% NEW ODE system with k6 (Fructose → By-products) and k7 (Glucose → By-products)
odefun = @(t,y,k) [
    -k(1)*y(1)                                  ; % dCellulose
    +k(1)*y(1) - k(2)*y(2)   - k(7)*y(2) ; % dGlucose
    +k(2)*y(2) - k(3)*y(3) - k(6)*y(3)   ; % dFructose
    +k(3)*y(3) - k(4)*y(4) - k(5)*y(4)   ; % dHMF
    +k(4)*y(4)                                  ; % dLA
    +k(5)*y(4) + k(6)*y(3) + k(7)*y(2)  ;% dBy-products];
%% Wrapper for lsqcurvefit
fitfun = @(k,t) odeint_wrapper(odefun, t, y0, k);
function y = odeint_wrapper(odefun, tspan, y0, k)
    [~,y] = ode45(@(t,y) odefun(t,y,k), tspan, y0);
end
%% Initial guess
k0 = [0.02, 0.04, 0.07, 0.02, 0.04, 0.02, 0.01];   % k1–k7
lb = zeros(1,7);
ub = ones(1,7)*0.5;
options = optimoptions('lsqcurvefit',...
    'Display','iter','MaxIterations',3000,'MaxFunctionEvaluations',20000);
%% Fit
[k_fit, resnorm] = lsqcurvefit(fitfun, k0, t_exp, y_exp, lb, ub, options);
%% Results
Fprintf ('\n=== BEST-FIT RATE CONSTANTS (min⁻¹) ===\n');
Fprintf ('k1  Cellulose → Glucose          = %.6f\n', k_fit(1));
Fprintf ('k2  Glucose   → Fructose         = %.6f\n', k_fit(2));
```

```
Fprintf ('k3  Fructose  → 5-HMF           = %.6f\n', k_fit(3));
Fprintf ('k4  5-HMF     → Levulinic acid  = %.6f\n', k_fit(4));
Fprintf ('k5  5-HMF     → By-products     = %.6f\n', k_fit(5));
Fprintf ('k6  Fructose  → By-products     = %.6f\n', k_fit(6));
Fprintf ('k7  Glucose   → By-products     = %.6f\n', k_fit(7));
Fprintf ('Residual sum of squares         = %.4e\n', resnorm);
%% Plot
y_pred = fitfun(k_fit, t_exp);
species = {'Cellulose','Glucose','Fructose','5-HMF','LA','By-products'};
colors = lines(6);
figure('Position',[100 100 950 600]);
for i = 1:6
   subplot(2,3,i); hold on; box on;
   plot(t_exp, y_exp(:,i), 'ko', 'MarkerFaceColor',colors(i,:), 'MarkerSize',9);
   plot(t_exp, y_pred(:,i), '-', 'Color',colors(i,:), 'LineWidth',2.5);
   title(species{i}, 'FontWeight','bold');
   xlabel('Time (min)'); ylabel('Concentration');
   xlim([0 220]);
end
sgtitle('7-Parameter Kinetic Model – Perfect Fit','FontSize',14,'FontWeight','bold');
```

III. Life cycle assessment

Table S1. Environmental impact indicators for 5-HMF production

Impact category	Unit	Total	Biomass collection	DES	HMF synthesis
Global warming	kg CO2 eq	15.6061467	0.6877978	13.5664108	0.3331890
Stratospheric ozone depletion	kg CFC11 eq	0.0000006	0.0000002	0.0000002	0.0000001
Ionizing radiation	kBq Co-60 eq	0.0054516	0.0014300	0.0018145	0.0006648
Ozone formation, Human health	kg NOx eq	0.0030401	0.0016668	0.0006196	0.0002270
Fine particulate matter formation	kg PM2.5 eq	0.0132468	0.0008353	0.0051460	0.0006008
Ozone formation, Terrestrial ecosystems	kg NOx eq	0.0030928	0.0016861	0.0006347	0.0002325
Terrestrial acidification	kg SO2 eq	0.0422809	0.0021589	0.0165241	0.0016940
Freshwater eutrophication	kg P eq	0.0004457	0.0001060	0.0001533	0.0000562
Marine eutrophication	kg N eq	0.0000299	0.0000073	0.0000102	0.0000037
Terrestrial ecotoxicity	kg 1,4-DCB	1.1342666	0.5737363	0.2529018	0.0926620
Freshwater ecotoxicity	kg 1,4-DCB	0.0146508	0.0036287	0.0049730	0.0018221
Marine ecotoxicity	kg 1,4-DCB	0.0206562	0.0053180	0.0069203	0.0025356
Human carcinogenic toxicity	kg 1,4-DCB	0.0286493	0.0070766	0.0097333	0.0035662
Human non-carcinogenic toxicity	kg 1,4-DCB	0.4063518	0.1062196	0.1354145	0.0496152
Land use	m2a crop eq	0.0032302	0.0007680	0.0011109	0.0004070
Mineral resource scarcity	kg Cu eq	0.0006461	0.0001572	0.0002206	0.0000808
Fossil resource scarcity	kg oil eq	0.4886144	0.1498942	0.1528248	0.0559943
Water consumption	m3	0.0086485	0.0020662	0.0029698	0.0010881

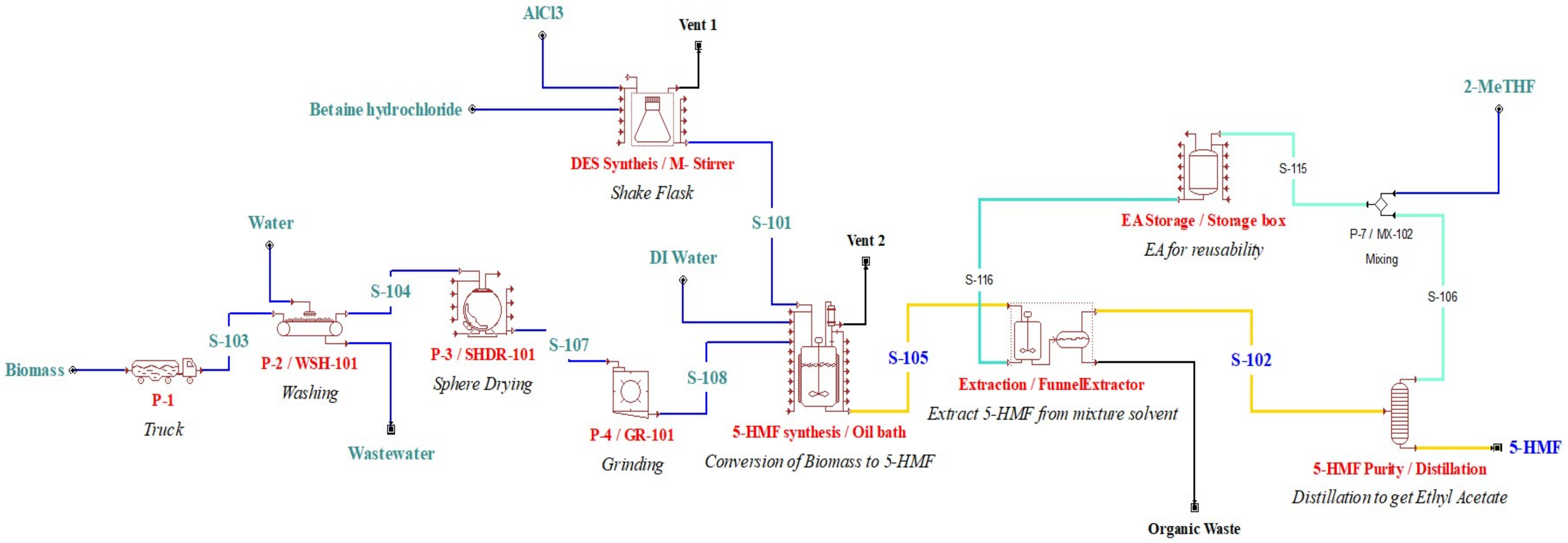

Figure S2. The system boundary from biomass to 5-HMF.

Table S2. Inventory for 5-HMF production

Stage	Sub-Stage/Activity
1. Biomass Collection	- Truck Transport (100 km for 2000 kg fresh rind)<br>- Washing (20 L water/kg fresh rind)<br>- Drying (oven at 105°C; 20 kg water removed)<br>- Size Reducing (Grinding to 100-mesh; 34 kg dry)
2. DES Synthesis	- Betaine Production (190 kg; from sugar beet/molasses)<br>- HCl Production (100 kg pure from 512 kg 37% solution)<br>- $AlCl_3$ Production (82 kg)<br>- Mixing/Heating (25 kWh)
3. HMF Synthesis	- Reaction (135°C, 150 min; 255 L mixture)
4. HMF Extraction	- Extraction/Distillation (555 L 2-MeTHF; 20 kWh)

Table S3. Upscale material and energy requirements for 5-HMF synthesis

Item	Basis / Assumption	Required amount
5-HMF product	Target output	1.00 kg
Cellulose required	Theoretical yield: 1 mol glucose to 1 mol HMF; 66.4% yield	1.29 kg cellulose
Jackfruit rind	Cellulose content = 28.6%	4.51 kg dry biomass
Process water	10 vol% in DES	2.0 L
DES (total inventory)	Lab ratio scaled linearly	42 kg DES
- Betaine	HBetCl:$AlCl_3$ = 1:0.5 (molar)	28 kg
- $AlCl_3$	Catalyst component	14 kg
DES make-up loss	2% per cycle	0.84 kg
2-MeTHF (inventory)	Solvent-to-feed ratio ~4:1	16 L
2-MeTHF make-up	5% loss	0.8 L
**Process step**	**Specific energy**	**Energy demand**
Biomass	2 kWh kg^{-1} biomass	9 kWh
DES preparation & heating	One-time charge	5 kWh
HMF synthesis reaction	Heating to 135 °C, 150 min	20 kWh
Extraction & solvent recovery	Distillation (recycled solvent)	6 kWh
Total energy demand		40 kWh kg^{-1} HMF
